# Characterization of *Penicillium oxalicum* SL2 isolated from indoor air and its application to the removal of hexavalent chromium

**DOI:** 10.1371/journal.pone.0191484

**Published:** 2018-01-30

**Authors:** Bibo Long, Binhui Ye, Qinglin Liu, Shu Zhang, Jien Ye, Lina Zou, Jiyan Shi

**Affiliations:** 1 Department of Environmental Engineering, Zhejiang University, Hangzhou, Zhejiang Province, China; 2 Key Laboratory for Water Pollution Control and Environmental Safety, Hangzhou, Zhejiang Province, China; Friedrich Schiller University, GERMANY

## Abstract

Removal of toxic Cr(VI) by microbial reduction is a promising approach to reducing its ecotoxicological impact. To develop bioremediation technologies, many studies have evaluated the application of microorganisms isolated from Cr(VI)-contaminated sites. Nonetheless, little attention has been given to microbes from the environments without a history of Cr(VI) contamination. In this study, we aimed to characterize the Cr(VI) tolerance and removal abilities of a filamentous fungus strain, SL2, isolated from indoor air. Based on phenotypic characterization and rDNA sequence analysis, SL2 was identified as *Penicillium oxalicum*, a species that has not been extensively studied regarding Cr(VI) tolerance and reduction abilities. SL2 showed high tolerance to Cr(VI) on solid and in liquid media, facilitating its application to Cr(VI)-contaminated environments. Growth curves of SL2 in the presence of 0, 100, 400, or 1000 mg/L Cr(VI) were well simulated by the modified Gompertz model. The relative maximal colony diameter and maximal growth rate decreased as Cr(VI) concentration increased, while the lag time increased. SL2 manifested remarkable efficacy of removing Cr(VI). Mass balance analysis indicated that SL2 removed Cr(VI) by reduction, and incorporated 0.79 mg of Cr per gram of dry biomass. In electroplating wastewater, the initial rate of Cr(VI) removal was affected by the initial contaminant concentration. In conclusion, *P*. *oxalicum* SL2 represents a promising new candidate for Cr(VI) removal. Our results significantly expand the knowledge on potential application of this microorganism.

## Introduction

Chromium (Cr) is a cause for considerable environmental concern because of its improper release into the environment from anthropogenic activities [1, 2]. Controlling the chemical state of Cr is vital for reducing its ecotoxicological impact. In nature, Cr mainly exists as Cr(VI) and Cr(III) compounds [3], which differ in their mobility and toxicity [4]. Cr(VI) compounds are water soluble in the full pH range and are toxic to humans [5, 6], animals [7], plants [8], and microorganisms [9], whereas Cr(III) compounds are less water soluble and serve as essential nutrients for energy metabolism [10]. Thus, reducing Cr(VI) to Cr(III) can minimize its harm to the environment and human health.

Various technologies have been developed to reduce Cr(VI) [11], including physicochemical and biological approaches [12]. Physicochemical remediation using functional materials such as polymers and nano materials is effective at Cr(VI) removal [13], however, most of them are expensive for large scale application and produce secondary environmental pollution. Alternatively, bioremediation by means of Cr(VI)-tolerant and -reductive microorganisms is considered particularly promising, and is eco-friendly and cost-effective [14]. Since Cr(VI) reduction by *Pseudomonas dechromaticans* was reported in the 1970s [15], many microorganisms with Cr(VI)-tolerant and -reductive properties have been isolated [16]. Nonetheless, most of these isolates are likely to be susceptible to Cr(VI) toxicity at higher concentrations, and have low efficacy of Cr(VI) removal [17], limiting their bioremediation applications. Hence, isolation of high-performance microorganisms is necessary to develop highly effective biological treatment technologies for Cr(VI) removal. Moreover, previous studies have mainly focused on Cr(VI)-tolerant and -reductive microorganisms isolated from Cr(VI)-contaminated sites [18], while little attention has been paid to microbes living without Cr(VI) selection pressure. To the best of our knowledge, no microorganisms have been isolated from indoor air for Cr(VI) removal. The isolation of Cr(VI)-tolerant and -reductive microorganisms from those environments with no history of Cr(VI) contamination may provide new candidates for Cr(VI) removal. Additionally, although fungi have certain advantages over bacteria [19], they have received less attention in studies on bioremediation of Cr(VI) contaminated environments. Hence, in the present study, we attempted to isolate a fungal strain from indoor air with the goal of characterizing its Cr(VI) tolerance and removal abilities via the modified Gompertz model and mass balance analysis. This study provides a new candidate for Cr(VI) removal, and the results significantly expand our knowledge on the utility of this microorganism.

## Experimental methods

### Ethics statement

No specific permits were required for the present study. Isolation of the Cr(VI)-tolerant fungus from indoor air of our work room did not cause any disturbance to the environment or involve protected species.

### Isolation of a Cr-tolerant microorganism

This fungal strain, which was named SL2, was isolated by a method similar to that previously employed for isolating Cr(VI)-tolerant fungi from air contaminated with industrial vapors [20]. The potato dextrose agar (PDA) solid medium was used for microorganism enrichment, and consisted of 1 g of dextrose, 1.8 g of agar, and 100 mL of filtered soup of 20 g of potatoes boiled for 30 min. The medium was autoclaved at 115°C for 20 min, cooled to approximately 60°C, supplemented with filter-sterilized potassium dichromate (300 mg/L) as a Cr(VI) source, and poured into dishes with 12 cm diameter. The dishes were placed in the open to collect potential Cr(VI)-tolerant and -reductive filamentous fungi from the indoor air of our work room in the Nongshenghuan Building at Zhejiang University in Hangzhou, Zhejiang, China (30°17'51.3"N 120°05'27.3"E). Upon growth and sporulation of the filamentous fungi, a spore suspension (SS) was prepared by rinsing the dishes in sterile water and then serially diluting the water 10-fold. The diluted SS (0.1 mL) was used to obtain single colonies by the spread plate method.

### Identification of the isolate

The isolated filamentous fungus was identified by phenotypic characterization and phylogenetic analysis. The latter was based on ribosomal DNA (rDNA; 18S rDNA, ITS, and 26S rDNA) amplification and sequence comparison. The primers for rDNA amplification are listed in Table 1. PCR was run as follows: pre-denaturation at 94°C for 10 min, then 32 cycles of denaturation at 94°C for 60 s, annealing at 55°C for 60 s, and polymerization at 72°C for 60 s (90 s for 18S rDNA). The obtained amplicon sequences were compared with published fungal sequences via BLAST (http://www.ncbi.nlm.nih.gov/blast) to identify the species of the isolate.

**Table 1:** Primers for the PCR amplification of rDNA.

rDNA	Primer name	Sequence
18S	NS1	5′-GTAGTCATATGCTTGTCTC-3′
	NS8	5′-TCCGCAGGTTCACCTACGGA-3′
ITS1	ITS1	5′-TCCGTAGGTGAACCTGCGG-3′
	ITS4	5′-TCCTCCGCTTATTGATATGC-3′
26S	NL1	5′-GCATATCAATAAGCGGAGGAAAAG-3′
	NL4	5′-GGTCCGTGTTTCAAGACGG-3′

### Growth of the isolate under Cr(VI) stress

The isolate was tested for Cr(VI) tolerance in PDA supplemented with different Cr(VI) concentrations. In accordance with another study that evaluated the heavy metal tolerance of fungi [21], the diameter of colonies was measured to quantify the growth of the isolate. The effect of Cr(VI) concentration on growth was analyzed by comparing the diameters of the fungal colonies. The PDA plates were subdivided into four sets and supplemented with 0, 100, 400, or 1000 mg/L Cr(VI), inoculated with 0.5 μL of SS, and incubated at 30°C. Cultures grown in the absence of Cr(VI) served as controls. The response of the isolate to Cr(VI) was expressed as a tolerance index, which was calculated as the mean diameter of the colonies exposed to Cr(VI) divided by that of the unexposed colonies [22].

Cr(VI) has been reported to have different toxic effects on microorganisms in a liquid medium as compared to a solid medium [23]. Thus, Cr(VI) tolerance of the isolate was assessed in a potato dextrose liquid medium (PDL) supplemented with different Cr(VI) concentrations. In keeping with the composition of PDA, PDL consisted of 1 g of dextrose and 100 mL of filtered soup of potato, and was autoclaved at 115°C for 20 min before use. PDL was divided into four aliquots and supplemented with 0, 100, 400, or 1000 mg/L Cr(VI). PDL without Cr(VI) served as the control. The groups were inoculated with SS and incubated in 250-mL conical flasks at 30°C in a rotary shaker (200 rpm). For each group, 3 replicates were filtered at regular intervals to obtain the mycelia, which were dried to a constant weight at 65°C in an oven for the analysis of dry biomass. The effects of Cr(VI) on growth were analyzed by comparing the dry biomass of the isolates grown in PDL containing different Cr(VI) concentrations. The tolerance index was calculated as the dry weight of the mycelia exposed to Cr(VI) divided by that of the control.

### Modeling the isolate’s growth curve under Cr(VI) stress

Growth curves of SL2 exposed to Cr(VI) were simulated by means of the modified Gompertz model, which is a three-parameter system and can describe microbial growth quantitatively [24]. The model can be expressed by the following equation [25]:

$$y=A\exp\left\{-\exp\left[\frac{\mu_{m}e}{A}\left(\lambda -t\right)+1\right]\right\}$$ (1)

where *y* is the relative colony diameter, *t* is time, and the three parameters, *A*, *μ*_*m*_, and *λ* are the relative maximal colony diameter, maximal growth rate, and lag time, respectively. *A* and *μ*_*m*_ indicate the growth of a microorganism, and *λ* means the time required for the microorganism to get to the reproductive stage [26].

The data for this analysis were obtained from the Cr(VI) tolerance test in PDA as described above. To train and validate the model, the diameters of the colonies grown at each Cr(VI) concentration were subdivided into a training data set and a validation data set. The training set consisted of diameters obtained on days 2, 3, 4, 6, 7, 8, 9, 11, 12, 13, 14, 17, 19, and 21. The validation set consisted of diameters obtained on days 5, 10, 15, and 20. The performance of the model was assessed by the coefficient of determination (*R*^*2*^), mean squared error (MSE), and Nash–Sutcliffe efficiency coefficient (NS). *R*^*2*^ measures the correlation between the experimental data and predicted values; MSE indicates the discrepancy between the measured and calculated values; and NS provides information on the predictive capability of the model. A perfect fit between the measured and predicted values would look like *R*^*2*^ = 1.0, MSE = 0, and NS = 1.0 [27].

### Scanning electron microscopy (SEM) analysis

SEM is useful for characterizing materials with an application potential in pollutant removal [28, 29]. In the present study, SEM analysis was carried out to detect morphological changes of the isolate when grown in PDL supplemented with 100 or 1000 mg/L Cr(VI). PDL cultures were inoculated with SS and incubated in 250 mL conical flasks at 30°C in a rotary shaker (200 rpm). A culture without Cr(VI) served as a control. After incubation for 48 or 144 h, fungal mycelia were collected and processed for SEM examination according to another study [30]. Details of the processing method are described in Supplementary Information. The processed mycelia were imaged at 20 kV under an SU-8010 Ultra-High-Resolution scanning electron microscope (Hitachi Corp., Tokyo, Japan).

### Mass balance of Cr(VI) removal

Because Cr(VI) may be removed by fungi through uptake and/or reduction [31], mass balance analysis was carried out to identify the fate of Cr(VI) after incubation with the isolate in PDL containing 199.6 mg/L Cr(VI). The medium was inoculated with SS, and a culture without inoculum served as a control. All the cultures were incubated in 250-mL conical flasks at 30°C in a rotary shaker (200 rpm). At days 0–6, samples of the culture medium were collected and filtered for analysis of pH, Cr(VI), and total Cr content. The concentration of Cr(VI) was determined by the diphenylcarbazide method, which has a detection limit of 0.2 μg/L [32]. The concentration of total Cr was determined by flame atomic absorption spectrophotometry on an MKILM6 spectrophotometer (Thermo Fisher Scientific, Waltham, MA).

For analysis of Cr uptake by the biomass, mycelia were collected from cultures by filtration after cultivation, washed with deionized water, dried to a constant weight at 65°C in an oven, and acid digested [33]. The method for digesting mycelia was described elsewhere[34]. Triplicate dry mycelia were prepared, resuspended in concentrated HNO_3_, and left at 30°C for 30 min, then incubated at 60°C for 30 min and then at 120°C for 30 min on a heating panel. When the digested solutions cooled to room temperature, they were supplemented with 30% H_2_O_2_ and heated again at 120°C for 15 min. After cooling to room temperature, the digested solutions were transferred to 100-mL volumetric flasks for analysis of total Cr.

### Removal of Cr(VI) from electroplating wastewater

To explore the utility of SL2 for removing Cr(VI) from actual wastewater, we tested electroplating wastewater, collected from a factory in Jinhua, Zhejiang, China. The concentrations of metals in this electroplating wastewater are shown in Supplementary Information. Given that the initial contaminant concentration may influence Cr(VI) removal [35], the electroplating wastewater was divided into 3 aliquots and diluted with deionized water at different ratios. For cultivation of mycelia, the diluted electroplating wastewater was adjusted to pH 7.0 with NaOH and supplemented with modified PDL as nutrition. The modified PDL consisted of 2 g of dextrose and 100 mL of filtered soup of 40 g of potatoes boiled for 30 min. The processed electroplating wastewater was inoculated with SS, and incubated in 250-mL conical flasks at 30°C in a rotary shaker (200 rpm). At regular intervals, samples were collected and filtered for the analysis of Cr(VI).

### Data analysis

Statistical analysis was carried out in the SPSS 20.0 software (IBM Corp., Armonk, NY). The paired-sample *t* test was performed to evaluate the differences in colony diameter and the tolerance index of the isolate at different Cr(VI) concentrations. One-way analysis of variance was conducted to compare the initial rates of Cr(VI) removal by the isolate from electroplating wastewater at various dilutions. Differences with *p* < 0.05 were regarded as statistically significant.

## Results and discussion

### Isolation and identification of the fungal strain

**Fig 1:**
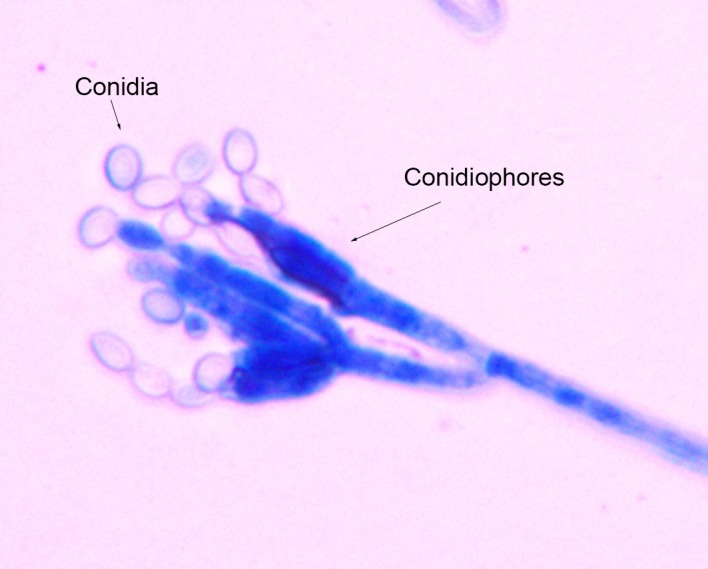
Microscopic examination of *Penicillium oxalicum* strain SL2, focusing on conidiophores and conidia (400× magnification).

A potential Cr(VI)-tolerant filamentous fungus strain, SL2, was isolated from indoor air and then purified in PDA supplemented with 300 mg/L Cr(VI). Colonies of SL2 grew rapidly and matured within 3 days of incubation at 30°C. The texture of its colony was velvety. The initial color was white, and gradually became dark green. The reverse color was dirty white. Microscopic observation showed that SL2 had septate hyphae, branched conidiophores, swollen phialides, and unicellular conidia (Fig 1). The conidia were oval, rough, and formed long chains. Sequence comparison revealed high similarities between the rDNA sequences (18S rDNA, ITS, and 26S rDNA) of SL2 and published *Penicillium oxalicum* sequences. Details are presented in Supplementary Information. According to phenotypic characterization and rDNA sequence analysis, SL2 was identified as *P*. *oxalicum*, a species that has not been extensively studied regarding Cr(VI) tolerance and reduction abilities.

Cr(VI) contamination is known to change the composition of microbial communities, including enrichment of microorganisms with elevated metal tolerance. Hence, most of the Cr-tolerant microorganisms reported in other studies have been isolated from Cr(VI)-contaminated sites [36, 37]. Unlike those microorganisms, *P*. *oxalicum* strain SL2 was isolated from an environment without a history of Cr(VI) contamination. The presence of Cr(VI)-tolerant microorganisms in indoor air provides evidence in support of these species being widespread in the environment. Thus, in addition to the microbiotas from sites contaminated by Cr(VI), microbial communities from sites without such contamination can harbor Cr(VI)-tolerant microorganisms holding promise for bioremediation.

### Effects of Cr(VI) concentration on strain SL2 growth

**Fig 2:**
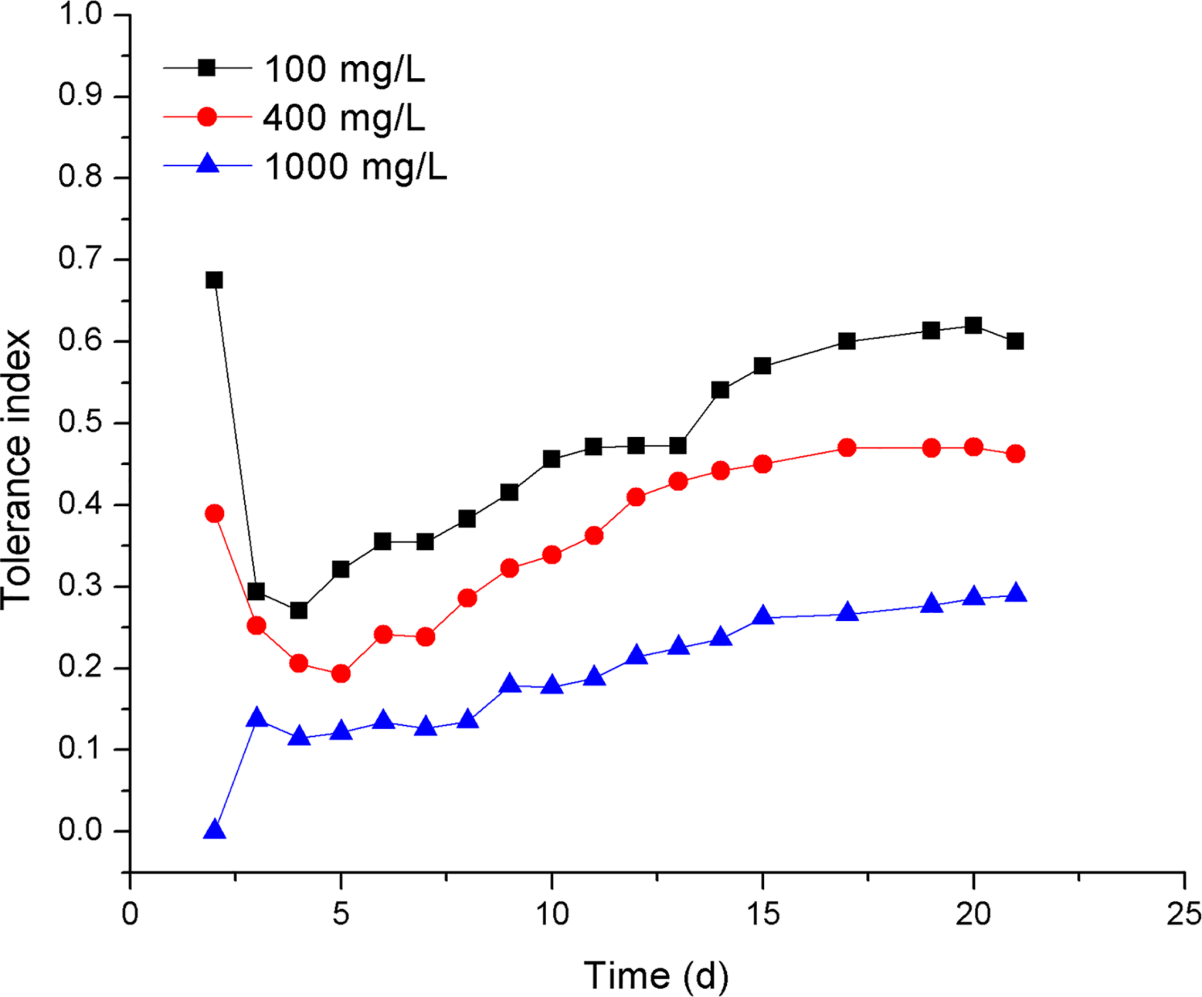
The tolerance index of SL2 in PDA solid media containing different concentrations of Cr(VI).

**Fig 3:**
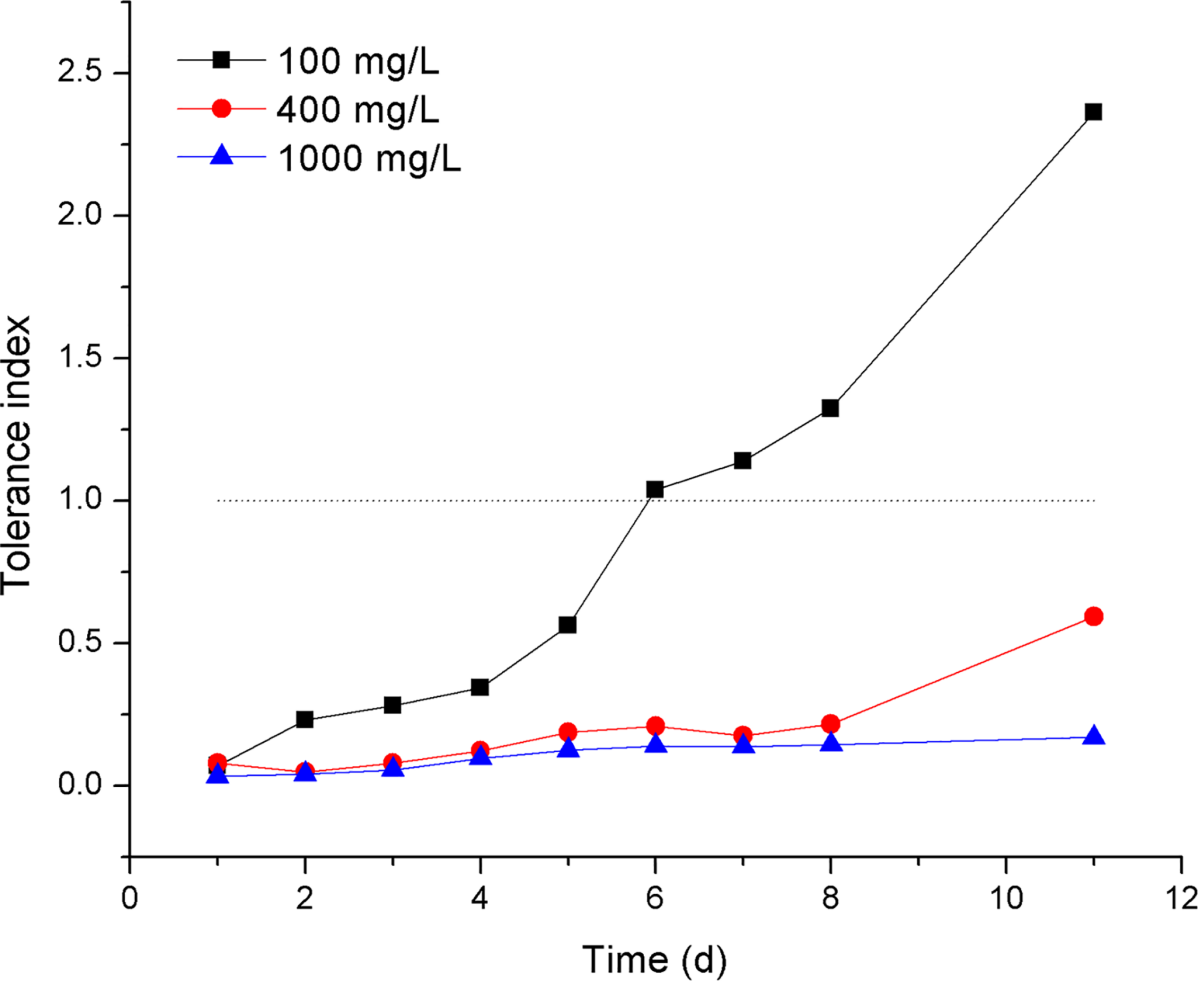
The tolerance index of SL2 in PDL media containing different concentrations of Cr(VI).

SL2 showed Cr(VI) tolerance in both PDA and PDL. SL2 grew after the inoculation of 0.5 μL SS onto PDA containing 1000 mg/L Cr(VI). Nevertheless, the diameters of SL2 colonies exposed to Cr(VI) were smaller than those in the unexposed control, resulting in tolerance indices less than 1.0 (Fig 2). Furthermore, the tolerance index of SL2 decreased with an increasing Cr(VI) concentration, indicating a negative relation between SL2 growth and Cr(VI) concentration in PDA. Besides, SL2 grew in PDL containing 0 mg/L, 100 mg/L, or 1000 mg/L Cr(VI). The differences between the dry weights of biomass of SL2 grown in PDL under 100 mg/L Cr(VI) stress and those in the control were not statistically significant (*p* = 0.11). In addition, after incubation for 6 days, SL2 developed more biomass when grown in PDL containing 100 mg/L Cr(VI) than in the control, which resulted in a tolerance index greater than 1.0 for SL2 grown under 100 mg/L Cr(VI) stress in PDL (Fig 3). This observation could be explained by SL2’s entering the death phase earlier in the control than at 100 mg/L Cr(VI), as a result, the biomass of SL2 in the control decreased and at the measurement time point was less than that in PDL containing 100 mg/L Cr(VI), which went into the death phase later. These results suggested that the growth of SL2 had different responses to Cr(VI) in liquid and solid media.

It has been reported that Cr(VI) has greater toxic effects on microorganisms in a liquid medium than on a solid one, thus leading to a difference in the minimum inhibitory concentration (MIC) of Cr(VI) [23, 38]. In the present study, SL2 was tolerant to 1000 mg/L Cr(VI) in PDA and PDL, implying that the MIC of Cr(VI) for SL2 is greater than 1000 mg/L on solid and in liquid media. Some studies indicate that Cr(VI)-tolerant microorganisms can survive in a wide range of Cr(VI) concentrations, but most of those microorganisms have a MIC less than 1000 mg/L [39]. Some Cr(VI)-tolerant microorganisms grow only at Cr(VI) concentrations of less than 100 mg/L [40–43]. Therefore, SL2 can tolerate a higher Cr(VI) concentration than most microorganisms can. Compared with other strains in the genus *Penicillium* [34], SL2 manifested distinctly higher tolerance to Cr(VI) as well. In general, SL2 showed high tolerance to Cr(VI) on solid and in liquid media, and this ability may facilitate its application to Cr(VI)-contaminated environments.

The modified Gompertz model was successfully applied to simulate the growth of strain SL2 under Cr(VI) stress. As shown in Table 2, the training data obtained at each of the tested concentrations fitted well to the modified Gompertz model, with *R*^*2*^_0mg/L_, *R*^*2*^_100mg/L_, *R*^*2*^_400mg/L_, and *R*^*2*^_1000mg/L_ close to 1.0. Moreover, the newly developed model was capable of predicting the growth curve of SL2 under Cr(VI) stress with good NS_0mg/L_, NS_100mg/L_, NS_400mg/L_, and NS_1000mg/L_ in the validation period. The good fit of the modified Gompertz model indicated that the growth curve patterns of SL2 were not significantly different with and without Cr(VI) stress. In addition, the newly developed model revealed that the growth of SL2 was affected by the Cr(VI) concentration (Table 3). The relative maximal colony diameter (*A*) and the maximal growth rate (μ_*m*_) decreased as Cr(VI) concentration increased from 0 to 1000 mg/L, while lag time (*λ*) increased, indicating that SL2 needed more time to adapt to greater Cr(VI) stress and get to the reproductive stage. As the modified Gompertz model successfully describes the growth of SL2 under Cr(VI) stress, it may be an effective tool for improving this microbe’s performance in practice.

**Table 2:** Performance statistics of the modified Gompertz model during training and validation periods for the modeling of growth curves of strain SL2 under Cr(VI) stress.

Cr(VI) concentration	Training			Validation		
	*R*^*2*^	MSE	NS	*R*^*2*^	MSE	NS
0 mg/L	0.996	0.023	0.996	0.984	0.078	0.975
100 mg/L	0.997	0.009	0.996	0.996	0.014	0.994
400 mg/L	0.990	0.018	0.989	0.991	0.020	0.987
1000 mg/L	0.993	0.004	0.993	0.986	0.008	0.986

*R*^2^, coefficient of determination; MSE, mean squared error; NS, Nash–Sutcliffe efficiency coefficient.

**Table 3:** Parameters of the modified Gompertz model for describing the growth of strain SL2 at different Cr(VI) concentrations.

Cr(VI) concentration	*A*	*μ*_*m*_	*λ*
0 mg/L	8.36	0.90	0.99
100 mg/L	5.65	0.37	1.92
400 mg/L	4.22	0.34	2.83
1000 mg/L	2.68	0.18	3.00

*A*, relative maximal colony diameter; *μ_m_*, maximal growth rate; *λ*, lag time.

### SEM analysis

**Fig 4:**
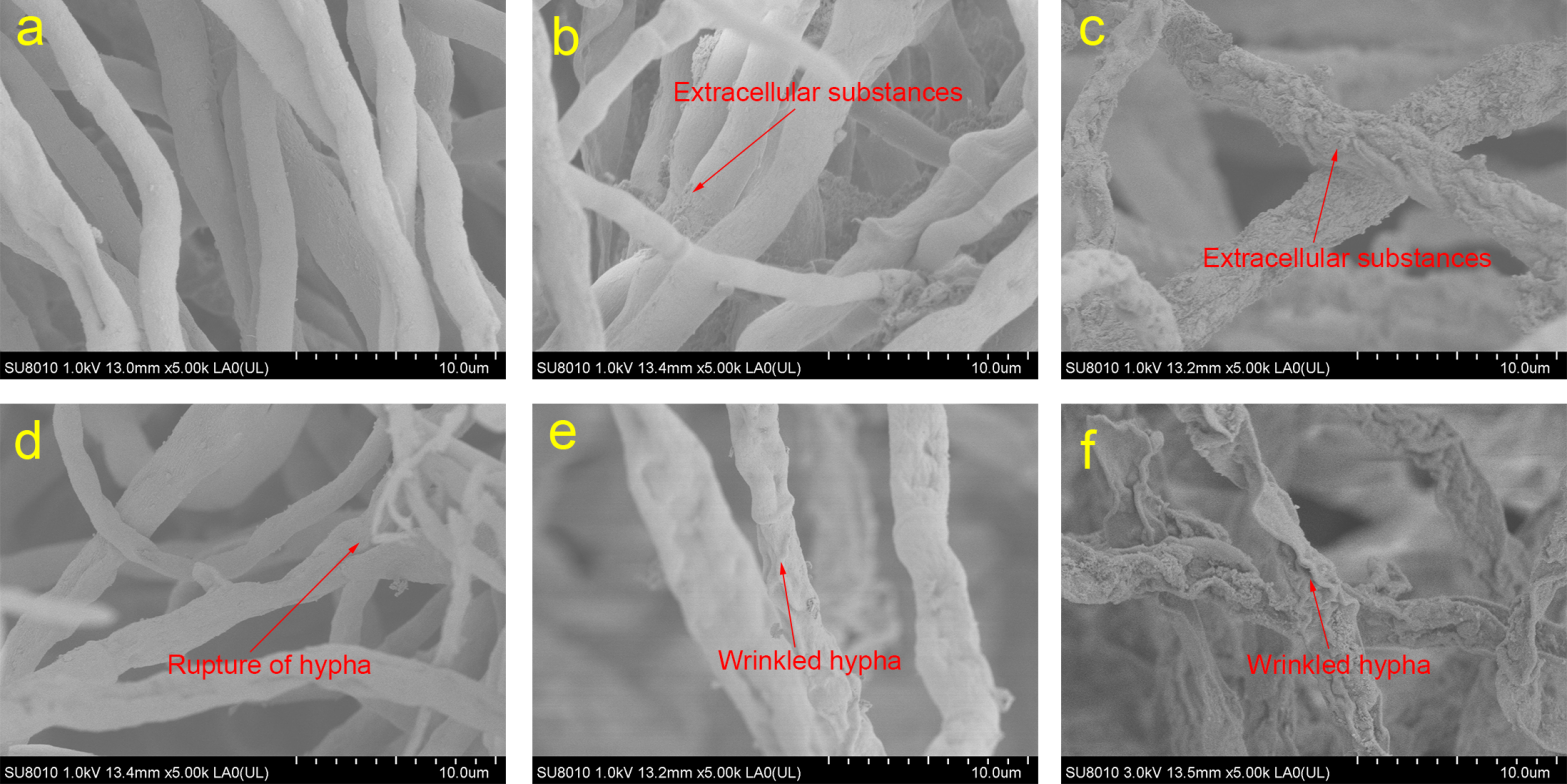
Micrographs of strain SL2 grown in PDL under Cr(VI) stress. Panels a, b, and c are images of strain SL2 grown for 48 h in the PDL medium containing 0, 100, or 1000 mg/L Cr(VI), respectively. Panels d, e, and f are images of SL2 grown for 144 h in the PDL medium containing 0, 100, or 1000 mg/L Cr(VI), respectively.

SEM micrographs of SL2 reveal the effects of Cr(VI) on its surface topography (Fig 4). After incubation for 48 h, strain SL2 grown without Cr(VI) stress had a regular shape, with few extracellular substances on the cell surface (Fig 4A). In contrast, strain SL2 grown under Cr(VI) stress showed increased amounts of extracellular substances on the cell surface (Fig 4B and 4C). In addition, SL2 grown under 1000 mg/L Cr(VI) stress had a relatively irregular shape. The results were in accordance with data from another study on an arbuscular mycorrhizal fungus that formed increased amounts of particles on its cell surface under Cr(VI) stress [44]. It was reported that alcohol, carboxyl, and amino groups may interact with heavy metals [45], and this interaction may result in their biosorption and flocculation [16, 46]. Hence, the production of extracellular substances containing these groups induced by Cr(VI) stress may be a strategy by which SL2 alleviates the poisonous effects of Cr(VI).

After incubation for 144 h without Cr(VI) stress, SL2 went into the death phase of its growth curve, and wrinkling and rupture of hypha were detected (Fig 4D). In the same period, hypha under 100 mg/L Cr(VI) stress were also wrinkled and irregular, but no rupture of hypha was detected in the 100 and 1000 mg/L Cr(VI) stress conditions (Fig 4E and 4F). These results supported the hypothesis that SL2 entered the death phase later under Cr(VI) stress than in the control condition. Moreover, the hypha of SL2 grown under 100 and 1000 mg/L Cr(VI) stress were bigger than those of strain SL2 grown without Cr(VI) stress. These results are consistent with those of other studies, which showed increases in the microbe size of some strains when exposed to Cr(VI) [47, 48]. The change in size of microorganisms could be a Cr(VI) stress response, allowing a strain to adapt to its environment.

### Mass balance of Cr(VI) removal

**Fig 5:**
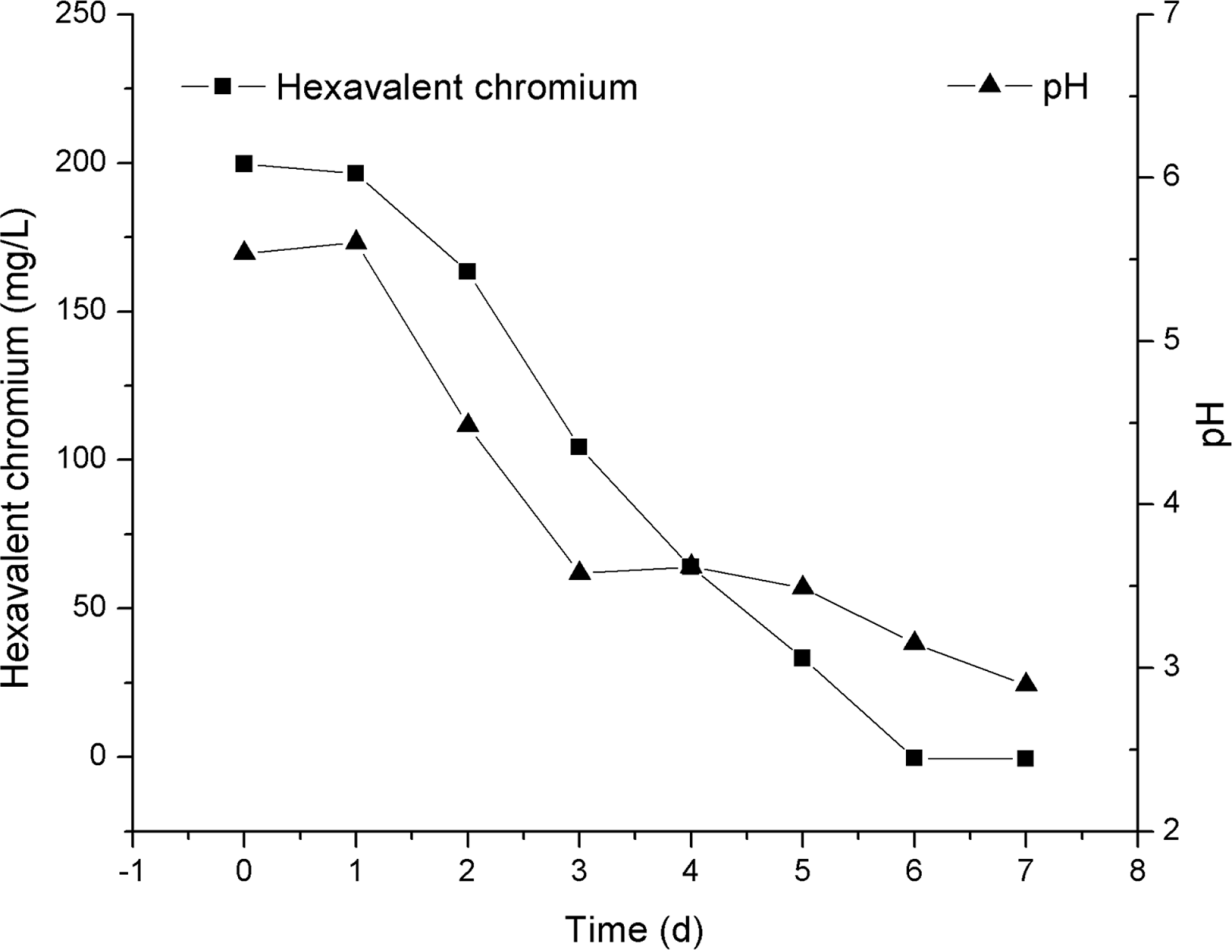
Cr(VI) removal and pH change during the incubation of SL2 in the PDL medium.

Cr(III) and Cr(VI) are the stable forms of Cr found in the environment, and Cr(III) is more difficult to quantify than Cr(VI) or total Cr, hence, most of the studies characterize Cr(VI) reduction ability of microorganisms by measuring the decrease in Cr(VI) concentration [49, 50], and the production of Cr(III) is indicated by the difference between total Cr and Cr(VI). In the present study, the isolated strain manifested remarkable efficacy at lowering the Cr(VI) level in PDL (Fig 5). After incubation for 7 days, Cr(VI) concentration decreased from the initial value of 199.6 mg/L to an undetectable level. Meanwhile, total Cr content in the medium did not change significantly over time, suggesting that Cr(VI) was reduced to Cr(III) and it accounted for most of the decrease in Cr(VI) concentration in PDL. In addition, the color of the liquid medium changed from the yellow color of soluble Cr(VI) to a slightly turbid brownish color, which may have been caused by the chemical conversion of chromium compounds in PDL. Our results are in agreement with other studies on *Aspergillus* sp., *Penicillium* sp., and *Paecilomyces* sp. [34, 37], which can quantitatively reduce Cr(VI) to Cr(III) and cause color changes in the medium. As reduction of Cr(VI) to Cr(III) can minimize the harmful effects of Cr, the Cr(VI) reduction ability should make fungal strain SL2 useful for bioremediation.

Cr(III) can be oxidized to Cr(VI) by strong oxidants [51], hence, the generated Cr(III) needs to be further removed from this reduction system. As a culture medium is an organic-matter-rich environment, Cr(III) may not be fully immobilized by strain SL2 owing to the coordination caused by soluble small organic molecules such as amino acids and organic acids in the culture medium [52]. Organo-Cr(III) species resulting from the coordination of Cr(III) and organic molecules are intermediates in the Cr biogeochemical cycle, and may convert into a Cr(OH)_3_ precipitate over time [53], and then could be removed by filtration.

Cr uptake by the biomass of strain SL2 was found to be 0.79 mg/g dry mass, and it accounted for ~0.95% of total Cr initially present in PDL. Compared with *Pleurotus* sp. [54], magnetic carbon-iron nanoadsorbents [55], polyaniline/ethyl cellulose [56], polyaniline/carbon fabrics [57], magnetic carbons [58], magnetic carbon nanoadsorbents [59], polyethylenimine/ethyl cellulose [60], and extracellular polymeric substances-modified polyaniline nanocomposites [61], strain SL2 incorporated less Cr into its biomass. Nevertheless, strain SL2 incorporated more Cr than did *Aspergillus* sp. and *Penicillium* sp. grown in the presence of 50 mg/L Cr(VI) [34]. It is important to note that Cr uptake by microorganisms may vary among different environments [34, 62]. Consequently, Cr uptake by the biomass of strain SL2 may change in different environmental conditions.

pH influences the chemical speciation, solubility, and bioavailability of Cr(VI). Therefore, the change of pH in PDL during Cr(VI) removal was studied next. Cr(VI) reduction has been reported to be a proton consuming process [63]. Accordingly, pH should theoretically increase as Cr(VI) is reduced. In contrast, as shown in Fig 5, the removal of Cr(VI) was accompanied by a decrease in pH in this study. This effect could be explained as follows: strain SL2 produced acidic metabolites [22], which provided more protons than those consumed during Cr(VI) removal. In addition to providing proton for Cr(VI) reduction, the decreased pH provided a high protonation level of the biomass with lots of positive charges, which thereby attracted the negative Cr(VI) ion. During the incubation of SL2, pH in PDL decreased from 5.54 to 2.90, and this change might have influenced the growth and Cr uptake by the biomass. Nevertheless, to gain a comprehensive understanding of the influence of pH, further study is needed.

### Removal of Cr(VI) from electroplating wastewater

**Fig 6:**
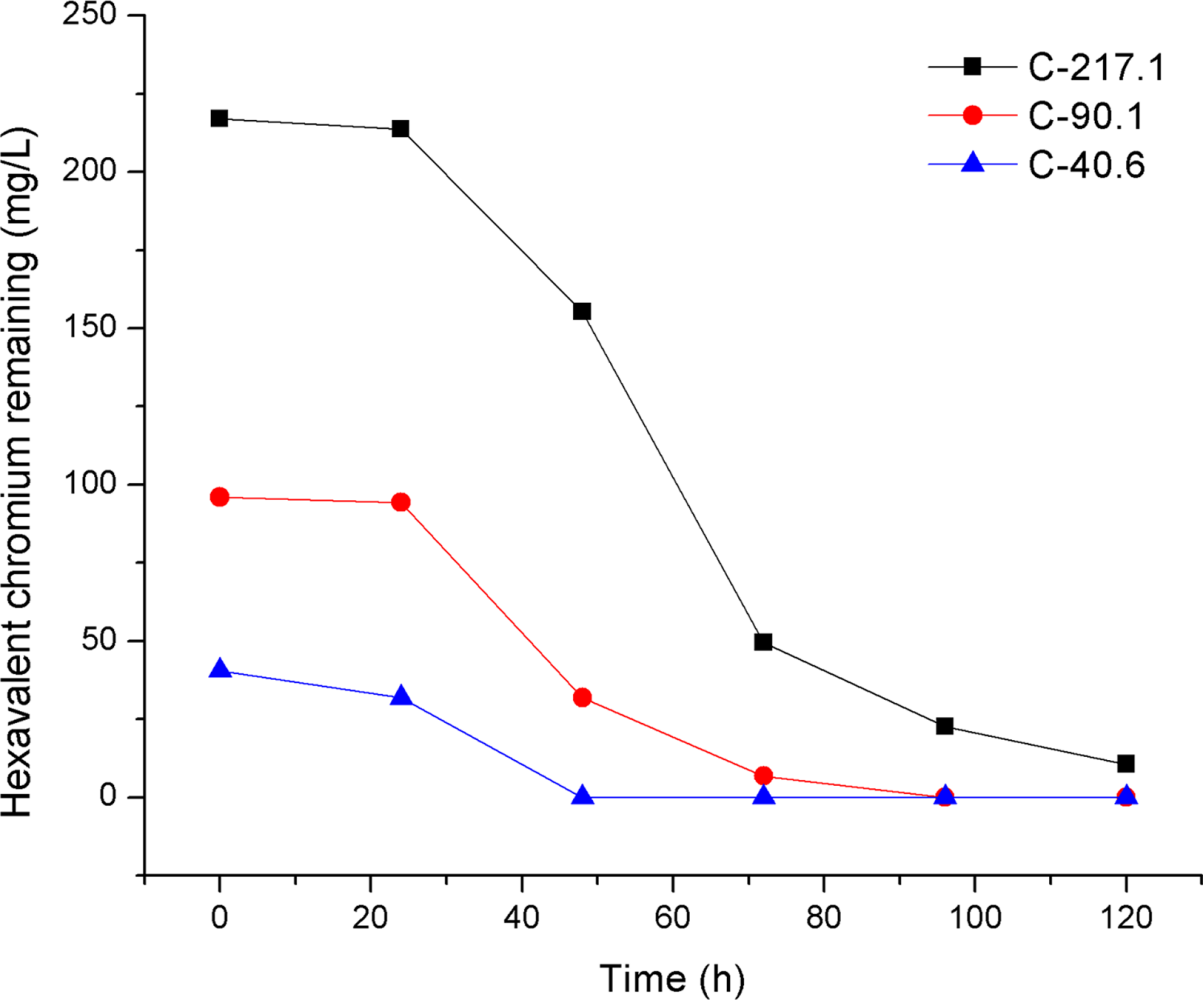
Cr(VI) removal by SL2 at different dilutions of electroplating wastewater. C-217.1, C-96.1, and C-40.6 represent treatments with initial Cr(VI) concentrations of 217.1, 96.1, and 40.6 mg/L, respectively.

**Fig 7:**
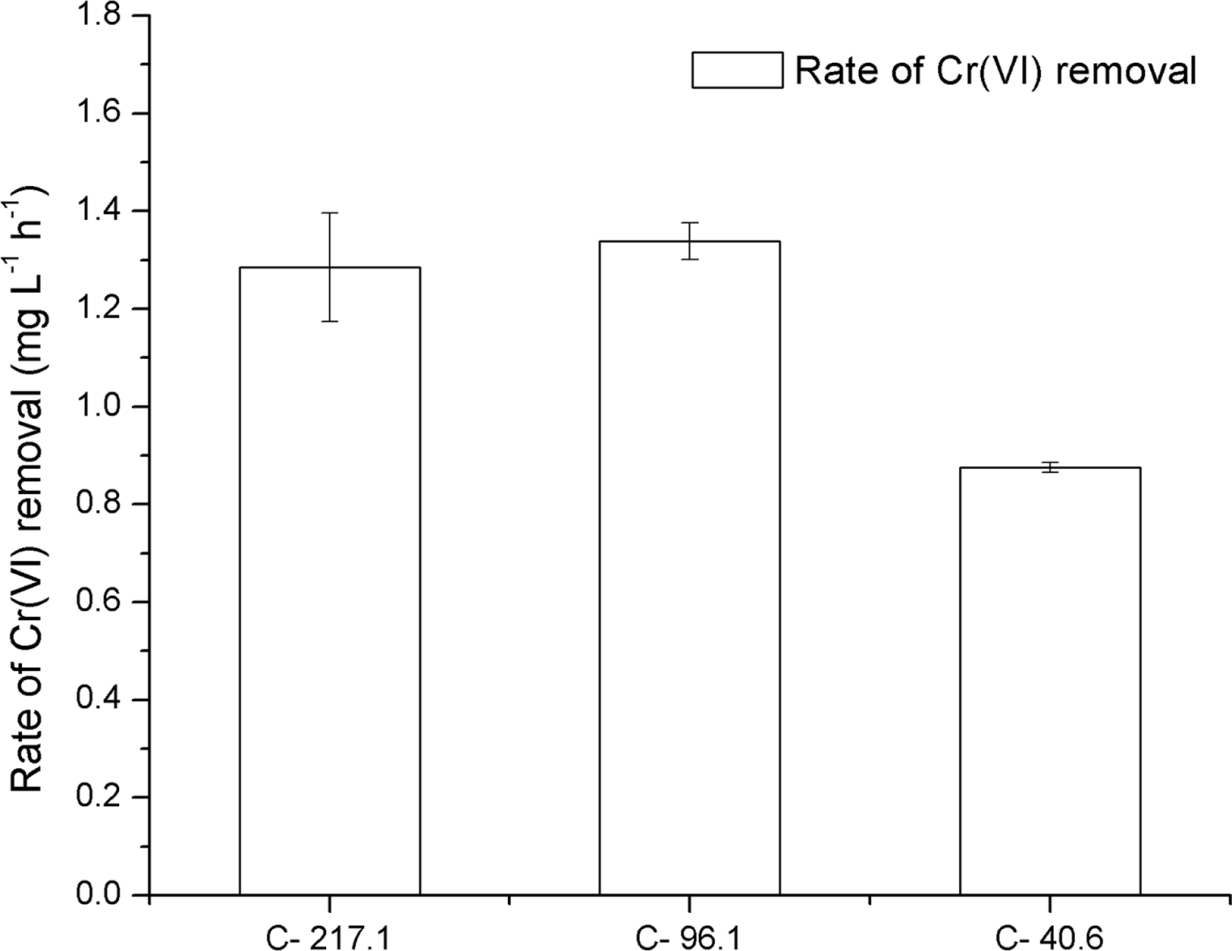
The initial rate of Cr(VI) removal by SL2 at different dilutions of electroplating wastewater. C-217.1, C-96.1, and C-40.6 represent treatments with initial Cr(VI) concentrations of 217.1, 96.1, and 40.6 mg/L, respectively. Error bars represent standard deviation. Treatments marked with the same letter are not significantly different.

Cr(VI) in electroplating wastewater was effectively removed by fungal strain SL2. As shown in Fig 6, after inoculation of spores of SL2, Cr(VI) in diluted electroplating wastewater at 40.6 and 96.1 mg/L was completely removed within 48 and 96 h, respectively. Furthermore, 89.6% of Cr(VI) in diluted electroplating wastewater at an initial concentration of 217.1 mg/L was removed in 96 h. The initial rate of Cr(VI) removal by SL2 was affected by the initial contaminant concentration. As depicted in Fig 7, Cr(VI) removal by SL2 was faster at 217.1 and 96.1 mg/L Cr(VI) in electroplating wastewater samples than in the sample with 40.6 mg/L Cr(VI), but there was no difference in the rate of Cr(VI) removal between the concentrations 217.1 and 96.1 mg/L. This phenomenon may be explained by the rate of collision of Cr(VI) with active sites on the cell surface [39]. The collision rate should increase as the initial contaminant concentration increases when the contaminant concentration was insufficient to inhibit the growth of the microorganism, and, as a result, the rate of Cr(VI) removal increased. In contrast, after the contaminant concentration exceeded the level that inhibits microbial growth, an increase in contaminant concentration decreased the number of active sites on the cell surface, as a result, the collision rate and Cr(VI) removal rate should not increase with an increase in contaminant concentration. Therefore, it is important to control the initial contaminant concentration when using SL2 to remove Cr(VI) from wastewater.

## Conclusion

Cr-tolerant microorganisms are widespread in the environment. Thus, in addition to microbiotas of Cr(VI)-contaminated sites, microbial communities in environments without Cr(VI) contamination can harbor Cr-tolerant microorganisms useful for Cr(VI) removal. *P*. *oxalicum* SL2, isolated from indoor air, was found to tolerate a high Cr(VI) concentration on solid and in liquid media, and this property may facilitate its application to Cr(VI)-contaminated environments. Simulated growth curves of SL2 exposed to Cr(VI) by means of the modified Gompertz model suggest that Cr(VI) decreases the relative maximal colony diameter and the maximal growth rate, and increases the period required for SL2 to adapt to Cr(VI) stress and get to the reproductive stage. SL2 showed remarkable efficiency to remove Cr(VI), and the mass balance suggests that this ion was reduced to Cr(III), but this process was not confirmed in this study. Our findings provide a new candidate for Cr(VI) removal, and will significantly expand our knowledge about the utility of this microorganism.

## Supporting information

S1 Table Metals in the electroplating wastewater samples having pH 1.8 and pH 7.0. (PDF)

S2 Table Comparison of strain SL2’s ITS with published fungal sequences using BLAST. (PDF)

S3 Table Comparison of strain SL2’s 26S rRNA gene with published fungal sequences by means of BLAST. (PDF)

S4 Table Comparison of strain SL2’s 18S rRNA gene with published fungal sequences via BLAST. (PDF)

S1 Text The method for processing of the mycelia for SEM analysis. (PDF)
