# Supplementary material for: Characterization of Penicillium oxalicum SL2 isolated from indoor air and its application to the removal of hexavalent chromium
Source: PLoS One. 2018 Jan 30;13(1):e0191484. doi: 10.1371/journal.pone.0191484 (PMC5790237; doi:10.1371/journal.pone.0191484)
Supplement: S1 Table — (PDF) [file pone.0191484.s001.pdf]

S1 Table. Metallic elements in the electroplating wastewater samples having pH 1.8 and pH 7.0

| pH | Metallic element (mg/L) | | | | | |
|---|---|---|---|---|---|---|
| | Fe | Mn | Ni | Zn | Ca | Cr |
| 1.8 | 95.5 | 0.7 | 23.3 | 41.3 | 13.7 | 409.2 |
| 7.1 | 0.1 | 0.2 | 4.5 | 2.42 | 11.4 | 300.2 |
