# Supplementary material for: Characterization of Penicillium oxalicum SL2 isolated from indoor air and its application to the removal of hexavalent chromium
Source: PLoS One. 2018 Jan 30;13(1):e0191484. doi: 10.1371/journal.pone.0191484 (PMC5790237; doi:10.1371/journal.pone.0191484)
Supplement: S2 Table — (PDF) [file pone.0191484.s002.pdf]

S2 Table. Comparation of strain SL2 ITS with published fungal sequences using BLAST

Accession	Description	Max score	Total score	Query cover	E value	Max ident
KF667524.1	Penicillium oxalicum strain WX-209 internal transcribed spacer 1, partial sequence; 5.8S ribosomal RNA gene and internal transcribed spacer 2, complete sequence; and 28S ribosomal RNA gene, partial sequence	1005	1005	100%	0	100%
JQ446378.1	Penicillium oxalicum isolate B3-11(2) 18S ribosomal RNA gene, partial sequence; internal transcribed spacer 1, 5.8S ribosomal RNA gene, and internal transcribed spacer 2, complete sequence; and 28S ribosomal RNA gene, partial sequence	1000	1000	100%	0	99%
HM044129.1	Penicillium oxalicum strain YQ1-2-1 internal transcribed spacer 1, partial sequence; 5.8S ribosomal RNA gene and internal transcribed spacer 2, complete sequence; and 28S ribosomal RNA gene, partial sequence	1000	1000	100%	0	99%
KF367495.1	Penicillium sp. 3 BRO-2013 18S ribosomal RNA gene, partial sequence; internal transcribed spacer 1, 5.8S ribosomal RNA gene, and internal transcribed spacer 2, complete sequence; and 28S ribosomal RNA gene, partial sequence	998	998	100%	0	99%
NR_121232.1	Penicillium oxalicum NRRL 787 ITS region; from TYPE material	998	998	100%	0	99%
KF152942.1	Penicillium oxalicum strain 114-2 18S ribosomal RNA gene, internal transcribed spacer 1, 5.8S ribosomal RNA gene, and internal transcribed	998	998	100%	0	99%

	spacer 2, complete sequence; and 28S ribosomal RNA gene, partial sequence					
KF358372.1	Penicillium oxalicum strain PJ02 internal transcribed spacer 1, partial sequence; 5.8S ribosomal RNA gene and internal transcribed spacer 2, complete sequence; and 28S ribosomal RNA gene, partial sequence	998	998	100%	0	99%
KC344971.1	Penicillium oxalicum strain a1s2_d38 18S ribosomal RNA gene, partial sequence; internal transcribed spacer 1, 5.8S ribosomal RNA gene, and internal transcribed spacer 2, complete sequence; and 28S ribosomal RNA gene, partial sequence	998	998	100%	0	99%
HM235946.1	Penicillium sp. 1-66 18S ribosomal RNA gene, partial sequence; internal transcribed spacer 1, 5.8S ribosomal RNA gene, and internal transcribed spacer 2, complete sequence; and 28S ribosomal RNA gene, partial sequence	998	998	100%	0	99%
