# Supplementary material for: Characterization of Penicillium oxalicum SL2 isolated from indoor air and its application to the removal of hexavalent chromium
Source: PLoS One. 2018 Jan 30;13(1):e0191484. doi: 10.1371/journal.pone.0191484 (PMC5790237; doi:10.1371/journal.pone.0191484)
Supplement: S3 Table — (PDF) [file pone.0191484.s003.pdf]

S3 Table. Comparation of strain SL2 26S rRNA gene with published fungal sequences using BLAST

Accession	Description	Max score	Total score	Query cover	E value	Max ident
KC759111.1	Penicillium oxalicum strain KUC1674 28S ribosomal RNA gene, partial sequence	1037	1037	100%	0	99%
HM469410.1	Penicillium oxalicum strain KUC1674 18S ribosomal RNA gene, partial sequence; internal transcribed spacer 1, 5.8S ribosomal RNA gene, and internal transcribed spacer 2, complete sequence; and 28S ribosomal RNA gene, partial sequence	1037	1037	100%	0	99%
FJ810802.1	Penicillium oxalicum 28S large subunit ribosomal RNA gene, partial sequence	1037	1037	100%	0	99%
AY213620.1	Penicillium oxalicum strain UWFP 974 28S ribosomal RNA gene, partial sequence	1033	1033	99%	0	99%
KF152942.1	Penicillium oxalicum strain 114-2 18S ribosomal RNA gene, internal transcribed spacer 1, 5.8S ribosomal RNA gene, and internal transcribed spacer 2, complete sequence; and 28S ribosomal RNA gene, partial sequence	1031	1031	100%	0	99%
AB438836.1	Soil fungal sp. DM2-336 gene for 28S rRNA, partial sequence	1027	1027	99%	0	99%
KF880949.1	Penicillium oxalicum strain 19.8 28S ribosomal RNA gene, partial sequence	1026	1026	100%	0	99%
JF812068.1	Penicillium oxalicum strain 2.1 26S ribosomal RNA gene, partial sequence	1026	1026	99%	0	99%

KF055408.1	Penicillium oxalicum strain P2V03 28S ribosomal RNA gene, partial sequence	1022	1022	97%	0	100%
KC341925.1	Fusarium sp. AL-19 IRH-2012f 26S ribosomal RNA gene, partial sequence	1020	1020	98%	0	99%
HE651152.1	Penicillium oxalicum genomic DNA containing 18S rRNA gene, ITS1, 5.8S rRNA gene, ITS2, 28S rRNA gene, culture collection CCF<CZE>:2315	1020	1020	97%	0	100%
HE651146.1	Penicillium oxalicum genomic DNA containing 18S rRNA gene, ITS1, 5.8S rRNA gene, ITS2, 28S rRNA gene, culture collection CCF<CZE>:2052	1011	1011	96%	0	100%
DQ840343.1	Penicillium sp. ZJ01 28S ribosomal RNA gene, partial sequence	1011	1011	96%	0	100%
