# Supplementary material for: Characterization of Penicillium oxalicum SL2 isolated from indoor air and its application to the removal of hexavalent chromium
Source: PLoS One. 2018 Jan 30;13(1):e0191484. doi: 10.1371/journal.pone.0191484 (PMC5790237; doi:10.1371/journal.pone.0191484)
Supplement: S4 Table — (PDF) [file pone.0191484.s004.pdf]

S4 Table. Comparation of strain SL2 18S rRNA gene with published fungal sequences using BLAST

Accession	Description	Max score	Total score	Query cover	E value	Max ident
KF152942.1	Penicillium oxalicum strain 114-2 18S ribosomal RNA gene, internal transcribed spacer 1, 5.8S ribosomal RNA gene, and internal transcribed spacer 2, complete sequence; and 28S ribosomal RNA gene, partial sequence	3085	3085	100%	0	100%
FJ458446.1	Penicillium decumbens strain ML-017 18S ribosomal RNA gene, partial sequence	3085	3085	100%	0	100%
EU667998.1	Penicillium decumbens strain 13-01 18S ribosomal RNA gene, partial sequence	3085	3085	100%	0	100%
EU273880.1	Penicillium decumbens strain L-06 18S ribosomal RNA gene, partial sequence	3085	3085	100%	0	100%
EU136028.1	Penicillium decumbens strain JU-A10 18S ribosomal RNA gene, partial sequence	3079	3079	100%	0	99%
KJ680324.1	Fungal sp. h2 18S ribosomal RNA gene, partial sequence	3073	3073	100%	0	99%
EF413620.1	Eupenicillium javanicum isolate AFTOL-ID 429 18S ribosomal RNA gene, partial sequence	3073	3073	100%	0	99%
U21298.1	Eupenicillium javanicum 18S small subunit rRNA gene	3073	3073	100%	0	99%
HQ455812.1	Penicillium decumbens strain C5 18S ribosomal RNA gene, partial sequence	3068	3068	100%	0	99%
GU082484.1	Penicillium decumbens strain ph-13 18S ribosomal RNA gene, partial sequence	3066	3066	100%	0	99%

AF548090.1	Penicillium glabrum strain ALI 218 18S ribosomal RNA gene, partial sequence	3048	3048	99%	0	99%
D88323.1	Penicillium malachiteum gene for 18S rRNA, partial sequence	3042	3042	99%	0	99%
FJ716242.1	Penicillium sp. 1F 18S ribosomal RNA gene, partial sequence	3040	3040	100%	0	99%
KC960012.1	Penicillium citrinum strain TG2 18S ribosomal RNA gene, partial sequence	3035	3035	100%	0	99%
KC790520.1	Penicillium sp. 8-12c 18S ribosomal RNA gene, partial sequence	3035	3035	98%	0	99%
HM161749.1	Penicillium sp. Y12 EG-2010 18S ribosomal RNA gene, partial sequence	3035	3035	100%	0	99%
EF395943.1	Penicillium sp. F2 18S ribosomal RNA gene, partial sequence	3035	3035	100%	0	99%
AB028190.1	Penicillium namyslowskii gene for 18S rRNA, partial sequence	3035	3035	100%	0	99%
KF758801.1	Penicillium citrinum strain Salicorn 46 18S ribosomal RNA gene, partial sequence	3029	3029	100%	0	99%
FR774046.1	Penicillium decumbens genomic DNA containing 18S rRNA gene, ITS1, 5.8S rRNA, ITS2, isolate MMH 89-p1	3027	3027	100%	0	99%
KC842215.1	Penicillium decumbens 16S ribosomal RNA gene, partial sequence	3025	3025	100%	0	99%
KC143067.1	Penicillium sp. 6-16M 18S ribosomal RNA gene, partial sequence	3025	3025	98%	0	99%
FR774045.1	Penicillium decumbens genomic DNA containing 18S rRNA gene, ITS1, 5.8S rRNA, ITS2,	3024	3024	100%	0	99%

	isolate ZHE 89-p3				
AB008406.1	Hemicarpenteles ornatus gene for 18S rRNA	3024	3024	100%	0	99%
