# Supplementary material for: Characterization of Penicillium oxalicum SL2 isolated from indoor air and its application to the removal of hexavalent chromium
Source: PLoS One. 2018 Jan 30;13(1):e0191484. doi: 10.1371/journal.pone.0191484 (PMC5790237; doi:10.1371/journal.pone.0191484)
Supplement: S1 Text — (PDF) [file pone.0191484.s005.pdf]

S1 Text. Processing method of the myceliums for TEM analysis

The myceliums were fixed in 2.5% glutaraldehyde at 4℃ overnight and rinsed for three times using 0.1 mM phosphate buffer (pH 7.0), then post-fixed in 1% osmic acid for 2h. Fixed myceliums were rinsed for three times using 0.1 mM phosphate buffer (pH 7.0), subsequently dehydrated using a graded ethanol seriers (50%, 70%, 80%, 90%, 95%, 100% ethanol) and dried with critical point drier (Emitech K850, England).
